# Molecular and biochemical responses of sesame (*Sesame indicum* L.) to rhizobacteria inoculation under water deficit

**DOI:** 10.3389/fpls.2023.1324643

**Published:** 2024-01-18

**Authors:** Anderson Reges dos Santos, Geisenilma Maria Gonçalves da Rocha, Alexandre Paulo Machado, Paulo Ivan Fernandes-Junior, Nair Helena Castro Arriel, Tarcisio Marcos de Souza Gondim, Liziane Maria de Lima

**Affiliations:** ^1^ Master’s Degree in Agricultural Sciences, State University of Paraiba (UEPB), Campina Grande, PB, Brazil; ^2^ Postdoc, Embrapa Algodão, Campina Grande, PB, Brazil; ^3^ Department of Basic Health Sciences, Federal University of Mato Grosso, Cuibá, MT, Brazil; ^4^ Embrapa Semiárido, Petrolina, PE, Brazil; ^5^ Embrapa Algodão, Campina Grande, PB, Brazil

**Keywords:** diazotrophic bacteria, symbiosis, antioxidant enzymes, *DREB1* and *HDZ7* genes, drought

## Abstract

**Introduction:**

Water scarcity is a challenge for sesame cultivation under rainfed conditions. In this scenario, a potential strategy to alleviate the water deficit is the application of plant growth-promoting bacteria. The objective of this study was to analyze the interaction of rhizobacteria with sesame cultivation under water deficit conditions.

**Methods:**

An experiment was conducted in pots in a greenhouse using the BRS Morena sesame cultivar. The experimental design was completely randomized in a factorial scheme: 2 (irrigation regimes - daily irrigation and water deficit by suspending irrigation until 90% stomatal closure) x 6 (treatments with nitrogen or inoculants), with 5 replications. The types of fertilization were characterized by the addition of nitrogen (ammonium sulfate; 21% N), inoculants based on Bacillus spp. (pant001, ESA 13, and ESA 402), Agrobacterium sp. (ESA 441), and without nitrogen (control). On the fifth day after the suspension of irrigation, plant material was collected for gene expression analysis (DREB1 and HDZ7), activities of antioxidant enzymes (superoxide dismutase and catalase), relative proline content, and photosynthetic pigments. At the end of the crop cycle (about 85 days), production characteristics (root dry matter, aboveground dry matter, number of capsules, and thousand seed weight), as well as leaf nitrogen (N) and phosphorus (P) content, were evaluated.

**Results and Discussion:**

There was a positive effect on both production and biochemical characteristics (proline, superoxide dismutase, catalase, and photosynthetic pigments). Regarding gene expression, most of the inoculated treatments exhibited increased expression of the DREB1 and HDZ7 genes. These biological indicators demonstrate the potential of rhizobacteria for application in sesame cultivation, providing nutritional supply and reducing the effects of water deficit.

## 1 Introduction

Sesame (*Sesamum indicum* L.) is an oilseed crop that has numerous nutritional benefits for humans and animals. Its seeds are rich in oil, proteins, carbohydrates, minerals, and various antioxidants (Dossou et al., 2023). The main antioxidants in sesame in terms of value and level are lignans; among lignans, sesamin and sesamolin are the main compounds due to their health-promoting properties (Abe-Kanoh et al., 2019; Majdalawieh et al., 2020).

Worldwide, the culture of sesame has great socioeconomic importance, the most recent data on seed production points to a production of 6.4 million tons, occupying 13 million hectares in 2021 (FAOSTAT, 2021).

Among various abiotic stresses, water deficit is one of the most detrimental and leads to significant reductions in crop yield (Wang et al., 2019). To mitigate the negative effects of drought, plants exhibit several physiological mechanisms that stem from the induction of various genes (Mehmood et al., 2022), antioxidant defense systems (Santos et al., 2022), and the accumulation of osmoprotectants (Lima et al., 2023), among other factors.

Understanding the physiological and molecular mechanisms induced by drought is a fundamental step toward the development of high-yielding and drought-tolerant crop varieties. Several crops such as maize (Sah et al., 2020), sugarcane (Pereira et al., 2022), and soybean (Dong et al., 2019) have long-standing research results reporting gene expression patterns under water stress conditions. Sesame genotypes tolerant to water deficit were also evaluated, and 61 candidate genes that confer greater tolerance to drought were identified. These genes may constitute useful resources for improving drought tolerance (Dossa et al., 2017).

Drought tolerance is a complex trait involving multiple genes associated with cellular signaling pathways that induce various physiological, morphological, and molecular responses. The membranes of plant cells perceive stress signals and trigger various self-activated and hormone-dependent signaling mechanisms, such as abscisic acid, for instance (Mahmood et al., 2019; Wang et al., 2022). Environmental signals related to drought are initially detected by specific receptors, which, upon activation, initiate metabolic pathways for intercellular signal transfer and activate transcription factors (TFs) for the expression of specific gene sets (Guttikonda et al., 2014). Transcription factors are among the most important gene families in the transcriptional regulation of responses to water stress (Dossa et al., 2016a; Dossa et al., 2016b; Baghery et al., 2022).

The homeodomain-leucine zipper (HD-Zip) gene family is involved in various stress-related processes in many plant species (Zhang et al., 2022) and plays a crucial role in protecting plants against biotic and abiotic stresses (Gao et al., 2015). HD-Zip is a well-characterized family of transcription factors found in several plant species. HD-Zip is associated with various regulatory processes in plant growth and development and responses to various external environmental signals; it is expressed in different plant tissues and organs and at various developmental stages (Wang et al., 2013; Capella et al., 2015; Ding et al., 2017).

Others genes are induced by drought. Transcription factors Dehydration-Responsive Element Binding (DREB1 and DREB2), belonging to the APETALA 2/Ethylene-Responsive Element Binding Protein (AP2/EREBP) group, bind to the dehydration-responsive element (DRE) cis-element in the promoter region of stress-inducible genes and regulate their expression in response to different abiotic stresses. The DREB subfamily plays a particularly important role in regulating plant responses to stress conditions (Dossa et al., 2016a; Zhang and Xia, 2023).

Recent studies have revealed that the application of plant growth-promoting bacteria (PGPB), like to the genera *Bacillus* spp. and *Bradyrhizobium* spp., can be used as a strategy to mitigate water stress (Brito et al., 2019; Lastochkina et al., 2019). The interaction between plants and microorganisms can also enhance the plant’s tolerance to water deficit, as already assessed in crops such as maize (Kavamura et al., 2013; Santos et al., 2020), sorghum (Santana et al., 2020), wheat (Gontia-Mishra et al., 2016), foxtail millet (Niu et al., 2018), tomato (Gowtham et al., 2020), and more recently in sesame (Lima et al., 2023). Inoculation with these bacteria can result in physiological changes such as the regulation of the antioxidant system (Barbosa et al., 2018; Andrade et al., 2021), stomatal closure regulation by altering abscisic acid levels (Chieb and Gachomo, 2023), and production of osmoregulators like trehalose and proline (Barbosa et al., 2018). For sesame cultivation, a pioneering study demonstrating PGPB as tools to reduce the effects of water stress was recently published by our research group (Lima et al., 2023). However, the mechanisms triggered by these bacteria in sesame remain unknown.

In the present study, the objective was to analyze the molecular response mechanisms to drought through the expression of the *DREB1* and *HDZ7* genes, along with the analysis of physiological and biochemical parameters, in the BRS Morena sesame cultivar inoculated with plant growth-promoting rhizobacteria and drought tolerance-promoting rhizobacteria.

## 2 Material and methods

### 2.1 Preparation of inoculants

The *Bacillus* spp. ESA 13 (Fernandes-Júnior et al., 2015), ESA 402 (Antunes et al., 2019), and *Agrobacterium* sp. ESA 441 (Silva et al., 2018) strains were deposited in the “Collection of Microorganisms of Agricultural Interest of Embrapa Semiárido” (Embrapa Semiárido, Petrolina-PE, Brazil). They were inoculated on solid Luria Bertani (LB) medium and incubated for 24 hours at 28 °C. Subsequently, they were subcultured in liquid LB medium and incubated at 28 °C, 180 rpm, for 72 hours until reaching the exponential phase of bacterial growth (1.0 x 10^9^ CFU ml^-1^) (Vincent, 1970). The inoculant containing the pant001 strain (Carvalho et al., 2023) (*Bacillus subtilis* - Panta Premium) was provided by the Geoclean company. Then the bacteria subcultured in liquid medium were used directly for seed inoculation.

### 2.2 Experiment procedure

The experiment was conducted in a greenhouse at Embrapa Algodão, Campina Grande, PB (latitude 07°13’ 30” S; longitude 35°54’17” W; altitude 546 m). The sesame cultivar BRS Morena was used in pots with a capacity of 20 liters, filled with sandy-clay soil. A soil sample was analyzed for physical and chemical characteristics (Teixeira et al., 2017), and based on the results, fertilization was carried out according to the crop’s demands (Gomes and Coutinho, 1998). Nitrogen was omitted in the treatments inoculated with bacteria and in the absolute control.

The experimental design was completely randomized in a factorial scheme of 2 (water regimes) x 6 (nitrogen treatments or inoculation), having a total of 12 treatments, with 5 replicates (**Table 1**). The water regimes were characterized as follows: 1) maintaining soil moisture with daily irrigation up to field capacity, and 2) inducing water deficit from the 30^th^ day after emergence (DAE) by suspending irrigation for five days when plants with wilting symptoms exhibited 90% stomatal closure. The duration of irrigation suspension was determined using an Infra Red Gas Analyzer (IRGA, model LCpro-SD). After the stress period, the plants were rehydrated, and water supply was resumed. The nitrogen treatment involved the application of ammonium sulfate, divided into two applications. The first application was made 15 days after emergence, and the second application was done 20 days after the first. The absolute control treatment did not receive any inoculation or nitrogen fertilization. The inoculated treatments included the application of inoculants containing one of the strains: pant001, ESA 13, ESA 402, or ESA 441.

**Table 1:** Treatments generated by the combination of factors (irrigation x sources of N) using BRS Morena sesame inoculated with rhizobacteria under water restriction.

No.	Treatments with irrigation	Concentration	No.	Treatments without irrigation	Concentration
1	Ammonium sulfate; phosphorus; potassium	24 g; 20 g; 7 g (per pot^-1^)	7	Ammonium sulfate; phosphorus; potassium	24 g; 20 g; 7 g (per pot^-1^)
2	Without N and without inoculation; phosphorus; potassium	(-); 20 g; 7 g (per pot^-1^)	8	Without N and without inoculation; phosphorus; potassium	(-); 20 g; 7 g (per pot^-1^)
3	pant001; phosphorus; potassium	1.0 x 10^9^ CFU ml^-1^; 20 g; 7 g (per pot^-1^)	9	pant001; phosphorus; potassium	1.0 x 10^9^ CFU ml^-1^; 20 g; 7 g (per pot^-1^)
4	ESA 13; phosphorus; potassium	1.0 x 10^9^ CFU ml^-1^; 20 g; 7 g (per pot^-1^)	10	ESA 13; phosphorus; potassium	1.0 x 10^9^ CFU ml^-1^; 20 g; 7 g (per pot^-1^)
5	ESA 402; phosphorus; potassium	1.0 x 10^9^ CFU ml^-1^; 20 g; 7 g (per pot^-1^)	11	ESA 402; phosphorus; potassium	1.0 x 10^9^ CFU ml^-1^; 20 g; 7 g (per pot^-1^)
6	ESA 441; phosphorus; potassium	1.0 x 10^9^ CFU ml^-1^; 20 g; 7 g (per pot^-1^)	12	ESA 441; phosphorus; potassium	1.0 x 10^9^ CFU ml^-1^; 20 g; 7 g (per pot^-1^)

Phosphorus and potassium fertilization followed the recommendation of Gomes and Coutinho (1998), using triple superphosphate containing in its composition 20% P_2_O_5_ and potassium chloride containing 60% K_2_O (**Table 1**).

The sesame seeds were disinfected with 70% ethanol for 15 seconds, 1% sodium hypochlorite for 1 minute, and rinsed 10 times with sterile distilled water (Hungria and Araujo, 1994). The disinfected seeds were placed in Petri dishes and microbiolized by immersion in the inoculants for 30 minutes. Ten seeds were sown per pot, and at 10 DAE of the seedlings, thinning was carried out, leaving only two plants per pot.

### 2.3 Vegetative development and productivity

The following growth characteristics were evaluated at the end of the phenological cycle (85 DAE): plant height (cm), measured from the base to the tip of the main stem of the plant; stem diameter; number of capsules per plant; mass of aboveground dry matter (g) and root dry matter (g), determined by drying the material in a forced-air circulation oven at 65 °C for approximately 96 hours until a constant mass was achieved, followed by weighing on a precision scale; and mass of 1000 seeds.

### 2.4 Analysis of nitrogen and phosphorus in the aboveground part

Initially, leaves from the plants at the end of the cycle were collected, placed in paper bags, and kept in a forced-air circulation oven for 72 hours at 65°C. After drying, the leaves were ground into a fine powder consistency using a knife mill.

For nitrogen analysis, a cold pre-digestion was performed at room temperature for 12 hours following the Kjeldahl method (Bezerra Neto and Barreto, 2011). Approximately 2 mg of dried plant material, 50 mg of sodium sulfate, 0.5 mg of copper sulfate, and 5 ml of sulfuric acid were used. Then, the solution was heated in a digestion block at 350 °C until all organic matter was dissolved, resulting in a clear solution. Subsequently, a 1 ml aliquot of the digested extract was added to a volumetric flask (50 ml) containing 40 ml of deionized water, 1 ml of sodium hydroxide, 1 ml of sodium silicate, and 2 ml of Nessler’s reagent. The volume was completed to 50 ml with deionized water. The reading was carried out in a spectrophotometer at 410 nm, and the accumulated nitrogen in the aboveground part was calculated according to Alcantara et al. (2014).

For phosphorus analysis, a solution containing 2 mg of dried plant material, 50 mg of sodium sulfate, 0.5 mg of copper sulfate, and 5 ml of sulfuric acid was prepared for cold pre-digestion at room temperature for 12 hours. Then, the solution was heated in a digestion block at 350 °C until all organic matter was dissolved, resulting in a clear solution. A 5 ml aliquot of the digested extract was added to a volumetric flask (50 ml) containing 10 ml of diluted molybdate solution and 40 mg of ascorbic acid and stirred until the mixture was homogeneous. The reading was carried out in a spectrophotometer at 660 nm. The phosphorus content in the samples was obtained from the relationship between absorbance value and concentration (Nogueira and Souza, 2005).

### 2.5 Enzymatic analyses and free proline content

A sample of two mature leaves from each plant was collected on the fifth day of water restriction (near 90% stomatal closure), immediately immersed in liquid N_2_, and stored at -80°C for subsequent protein extraction. For protein extraction, 0.2 g of the leaves were ground in liquid N_2_, and 3 ml of 0.1 M potassium phosphate buffer, pH 7.0, containing 100 mM EDTA, 1 mM L-ascorbic acid, and 4% polyvinylpyrrolidone (PVP) were added. Protein quantification was performed using the Bradford method (1976) on a spectrophotometer at 595 nm. The protein extract was stored at -20°C for enzymatic analyzes.

The activity of superoxide dismutase (SOD) was determined using the Giannopolitis and Ries method (1977) with 40 μl of leaf protein extract, 1.5 ml of 100 mM potassium phosphate buffer, pH 7.8, containing 1 mM EDTA, 13 mM methionine, 75 mM p-nitrotriazolium blue (NBT), and 460 μl of 1 mM riboflavin. The activity was determined using a spectrophotometer at 560 nm.

The catalase activity (CAT) was determined according to Azevedo et al. (1998). A 100 μl aliquot of leaf protein extract was mixed with 2.9 ml of 100 mM potassium phosphate buffer, pH 7.0, containing 40 mM H_2_O_2_. The reading was performed using a spectrophotometer at 240 nm.

The free proline content was determined using the methodology described by Bates et al. (1973). The reading was performed on a spectrophotometer at 520 nm.

### 2.6 Photosynthetic pigments

The levels of chlorophyll a, b, and total (a+b), as well as carotenoids, were determined using the 80% acetone extraction method (Lichtenthaler, 1987). The entire procedure was carried out in the presence of green light to prevent chlorophyll degradation. A 200 mg sample of plant tissue was ground in liquid N_2_ and then solubilized in 10 ml of 80% acetone. Subsequently, the solution was filtered through qualitative filter paper, and the following absorbances were determined: 470; 646.8; 663.2; and 710 nm.

### 2.7 Analysis of *HDZ7* and *DREB1* gene expression by RT-qPCR

For gene expression analyses, leaves were collected on the fifth day of water restriction (near 90% stomatal closure), immersed in liquid N_2_, and stored at -80°C. Total RNA was extracted using the Invisorb Spin Plant RNA Mini kit (Invitec). RNA integrity was assessed by 0.8% agarose gel electrophoresis, and concentration and purity were determined by spectrophotometry. A 1 µg aliquot of RNA was treated with DNase I, and cDNA synthesis was performed using the ImProm-II™ Reverse Transcription System kit (Promega). All procedures followed the manufacturers’ recommendations.

Real-time quantitative analyses were conducted on the QuantStudio™ 5 Real-Time PCR System thermocycler (ThermoFisher) to amplify fragments of approximately 180 bp at an annealing temperature of 60°C. For each reaction, 6 µl of GoTaq^®^ qPCR Master Mix (Promega), 0.4 µl of each primer (10 µM) (**Table 2**), 1.0 µl of cDNA (diluted 1:20, v/v), and water were used to achieve a final volume of 12 µl. The amplification conditions were as follows: 95°C for 10 minutes and 40 cycles of 95°C for 15 seconds, 60°C for 1 minute, and finally 72°C for 15 seconds. The graphs, melt curves, and Cqs were automatically generated by the QuantStudio™ 5 Real-Time PCR System thermocycler (ThermoFisher) based on the ΔΔCq normalization method (Livak and Schmittgen, 2001). Relative quantification was used for pattern analysis. Reactions with the constitutive actin gene were performed as an endogenous control.

**Table 2:** Genes and sequences of the oligonucleotides used in quantitative analyses.

Gene	Sequence 5’→3’	Reference
*HDZ7*	F- TCTAAGCAAATCGAGCAAGAG R- TCTGTCTACCTGGGTGAGC	Mehmood et al. (2022)
*DREB1*	F- AGGGAGCCCAACAAGA R- TTAGCATTCGCAGACG	Dossa et al. (2016a)
*ACTINA*	F- CTGTCAACAGAATTGGGTG R- GCAACTGGGATGATATGG	Wei et al. (2015)

### 2.8 Statistical analysis

The collected data was analyzed using the statistical software SISVAR version 5.6 (Ferreira, 2014), subjected to analysis of variance, and means were compared using the Tukey test (P ≤ 0.05).

## 3 Results

### 3.1 Vegetative growth and grain production

**Figure 1:**
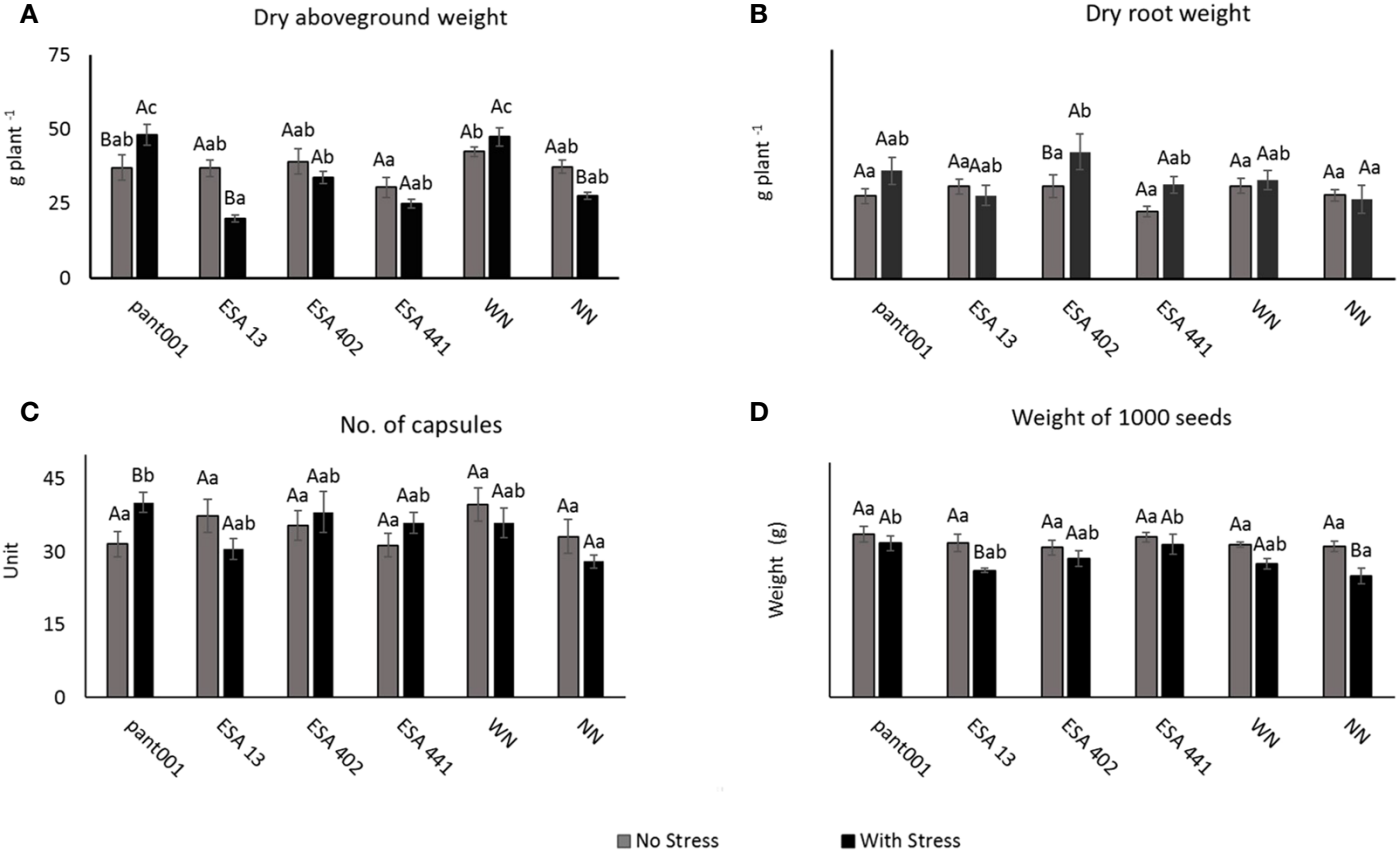
**(A)** Dry aboveground biomass; **(B)** Dry root weight; **(C)** Number of capsules; **(D)** Weight of a thousand sesame seeds of BRS Morena sesame inoculated with rhizobacteria under water restriction. Lowercase letters compare the treatments; uppercase letters compare the water regimes within each treatment. With nitrogen - WN; Without nitrogen – NN; No stress - daily irrigation; With stress - suspension of irrigation for five days, with stomatal closure near 90%. Tukey at 5% probability.

Regarding the dry aboveground biomass, under water stress, plants inoculated with pant001 showed the highest average compared to the other treatments, followed by plants with nitrogen (WN) and inoculated with ESA 402 (**Figure 1A**). About the dry root mass production, a significant effect was observed in the ESA 402 treatment (**Figure 1B**). For the number of capsules, the pant001 treatment under water stress obtained a higher average with a 21% increase compared to the irrigated treatment (**Figure 1C**). When analyzing the mass of a thousand seeds, it was observed that the pant001 and ESA 441 treatments under water stress had the highest averages compared to the non-inoculated treatments (**Figure 1D**).

### 3.2 Analysis of nitrogen and phosphorus in plant tissue

**Figure 2:**
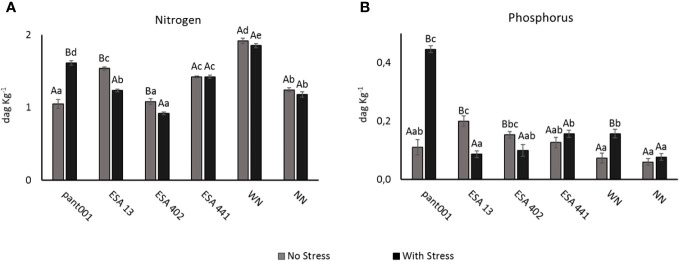
Accumulation of nitrogen **(A)** and phosphorus **(B)** in leaves of BRS Morena sesame inoculated with rhizobacteria under water restriction. Lowercase letters compare the treatments; uppercase letters compare the water regimes within each treatment. With nitrogen - WN; Without nitrogen – NN; No stress - daily irrigation; With stress - suspension of irrigation for five days, with stomatal closure near 90%. Tukey at 5% probability.

When analyzing the nitrogen content in leaves at the end of the experiment, the nitrogen-fertilized treatment, in both water conditions, had higher averages. Another treatment that showed a significant increase in nitrogen content was pant001 under water stress, compared to the irrigated condition, with an increase of 34% (**Figure 2A**). Regarding phosphorus content, the treatments with pant001, ESA 13, ESA 402, and nitrogen showed statistical differences when comparing the water conditions. Non-irrigated plants inoculated with pant001 had a higher concentration (0.45 dag kg^-1^), an increase of 75%, compared to their irrigated treatment (**Figure 2B**).

### 3.3 Proline content and antioxidant activities

**Figure 3:**
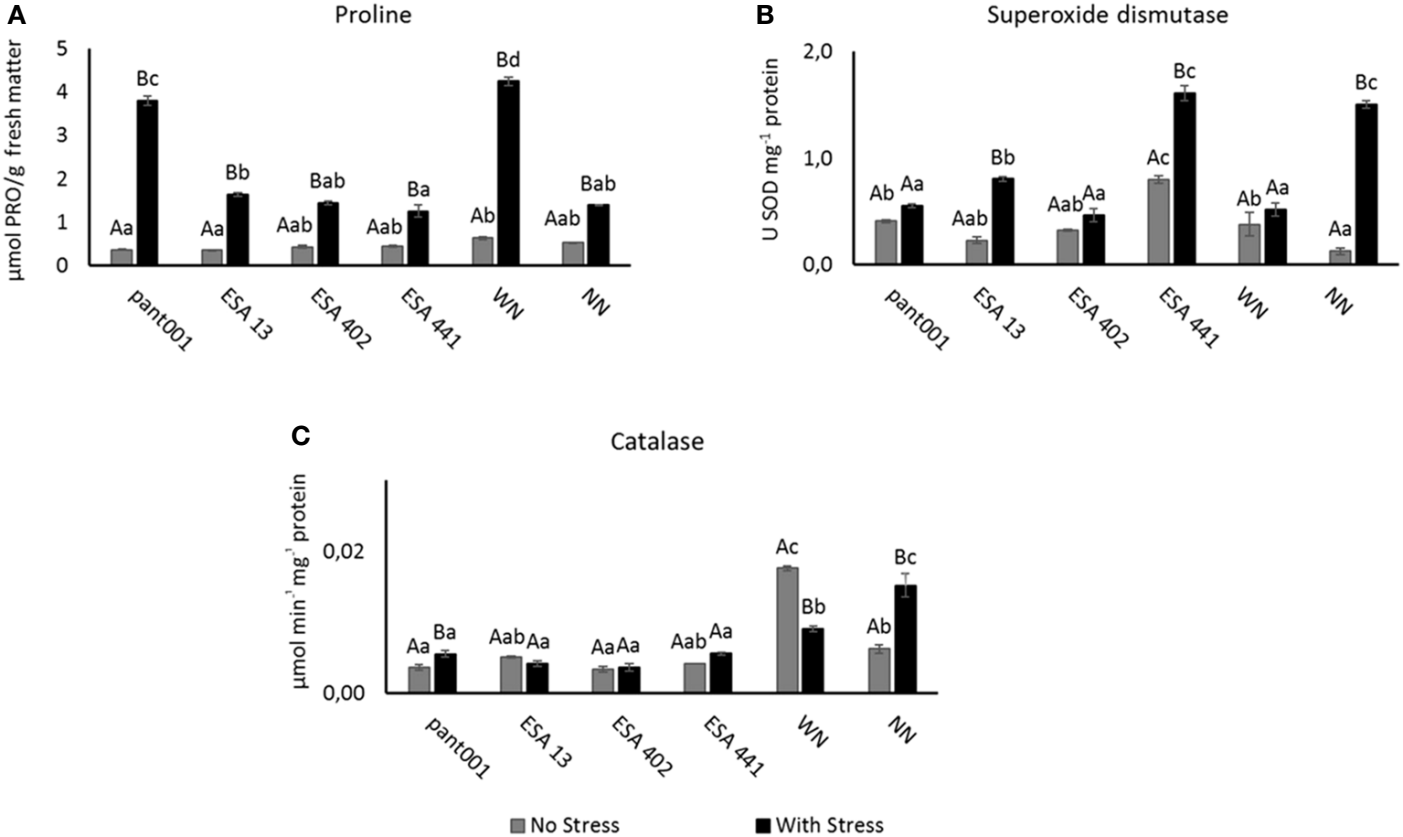
Analysis of sesame plants inoculated with rhizobacteria and under water restriction: **(A)** Concentration of free proline; **(B)** superoxide dismutase - SOD; **(C)** catalase - CAT. Lowercase letters compare the treatments; uppercase letters compare the water regimes within each treatment. With nitrogen - WN; Without nitrogen – NN; No stress - daily irrigation; With stress - suspension of irrigation for five days, with stomatal closure near 90%. Tukey at 5% probability.

In the analysis of free proline, an increase in the concentration of this amino acid was observed in all treatments under water deficit conditions, differing statistically from the irrigated condition. Stressed plants treated with pant001, as well as those fertilized with nitrogen, had a higher concentration of proline, corresponding to an increase of 63% and 67%, respectively, when compared to plants without nitrogen (NN) under water stress (**Figure 3A**).

The analysis of SOD activity revealed a higher concentration in treatments under water stress, implying a physiological adjustment of the plants to water deficit, with significant differences between these water conditions. Plants inoculated with ESA 441 under water stress had higher SOD activity, with an increase of 50% when compared to the irrigated condition (**Figure 3B**). CAT activity, under water stress, differed statistically from the irrigated condition in treatments with pant001 and NN (**Figure 3C**).

### 3.4 Photosynthetic pigments

**Figure 4:**
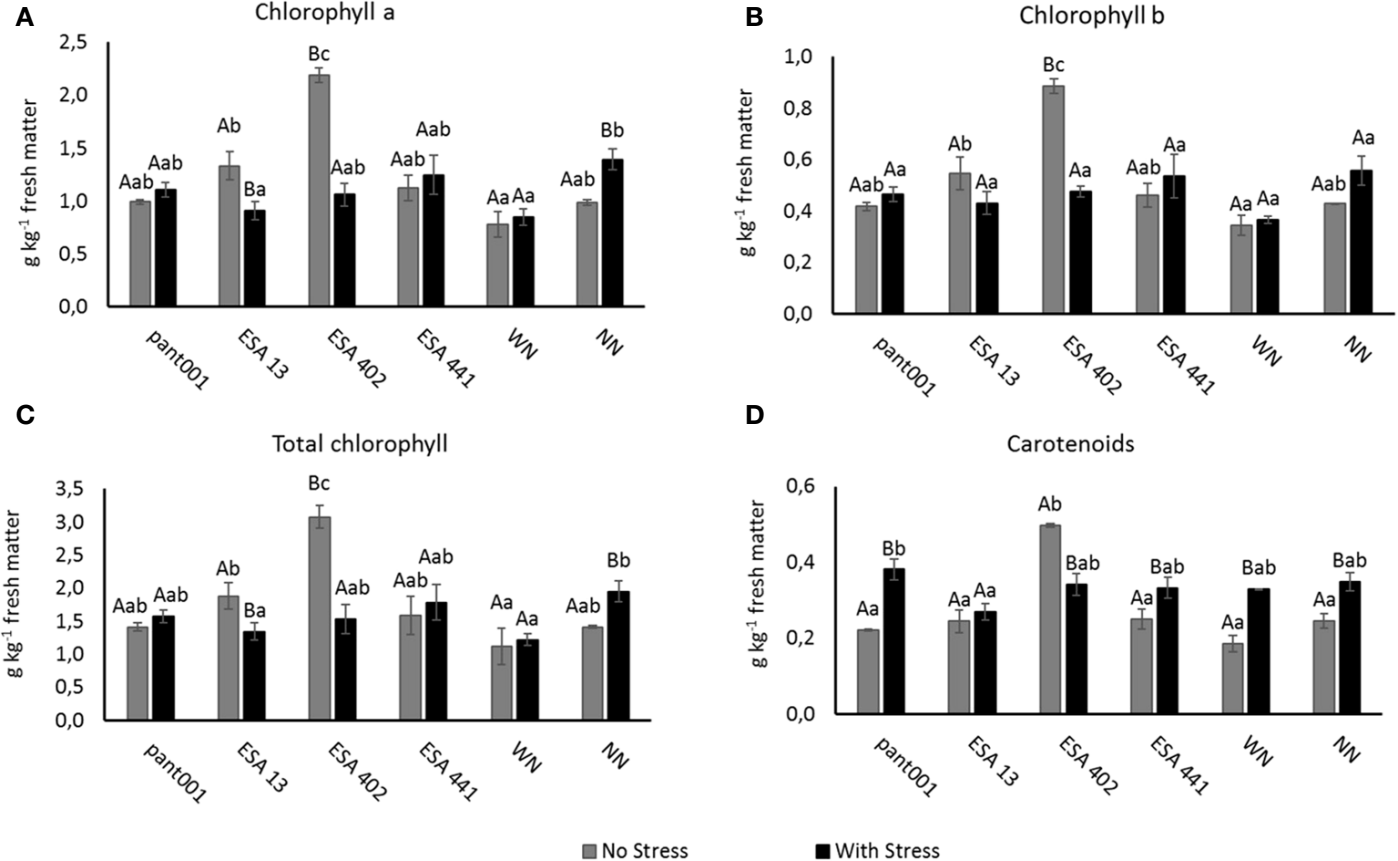
Analysis of photosynthetic pigments in sesame plants BRS Morena, inoculated with rhizobacteria and under water restriction: **(A)** chlorophyll a; **(B)** chlorophyll b; **(C)** total chlorophyll; **(D)** carotenoids. Lowercase letters compare the treatments; uppercase letters compare the water regimes within each treatment. With nitrogen - WN; without nitrogen - NN; No stress - daily irrigation; With stress - suspension of irrigation for five days, with stomatal closure near 90%. (Tukey at 5% probability).

Despite the water restriction, treatments with pant001, ESA 441, WN, and NN showed a positive interaction, increasing the concentration of chlorophyll a, b, and total chlorophyll (**Figures 4A–C**, respectively). Under irrigated conditions, the best treatment for both forms of chlorophyll, as well as total chlorophyll, was with ESA 402, with an increase of approximately 55% compared to the treatment without nitrogen.

Regarding the concentration of carotenoids (**Figure 4D**), it was observed that most of the plants under water deficit increased the production of this pigment, with a significant difference between the water conditions. The treatment with pant001 showed the highest average increase when compared to the other non-irrigated treatments.

### 3.5 Expression of *HDZ7* and *DREB1* Genes

**Figure 5:**
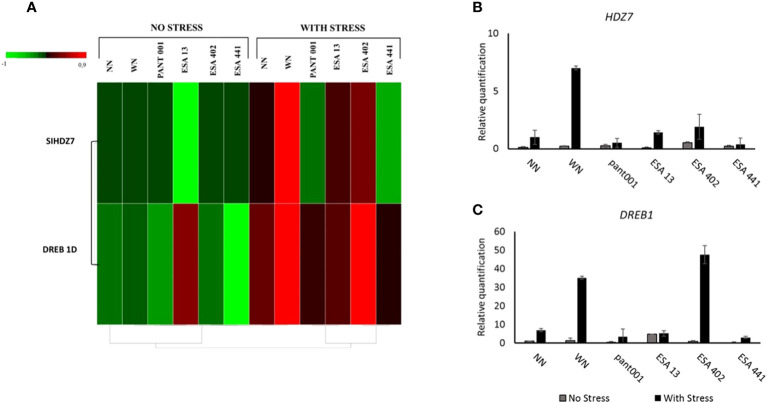
Analysis of the expression profile of the *HDZ7* and *DREB1* genes in the leaf tissues on the 5^th^ day of water deficit (90% stomatal closure) in BRS Morena sesame plants inoculated with rhizobacteria. **(A)** Gene expression heatmap highlighting the difference in expression for plants without water stress versus plants under water deficit; Quantitative expression of the HDZ7 **(B)** and DREB1 **(C)** genes. With nitrogen - WN; without nitrogen – NN; No stress - daily irrigation; With stress - suspension of irrigation for five days, with stomatal closure near 90%. Tukey at 5% probability.

The *HDZ7* gene was significantly expressed in all stressed plants (**Figures 5A, B**). Plants WN had higher expression, being seven times higher than nitrogen-free plants. The treatments with ESA 402 and ESA 13 were also highlighted.

All plants under water deficit conditions showed an induction of the *DREB1* gene expression. Plants inoculated with ESA 402 exhibited higher expression, being eight times higher than the non-irrigated treatment without nitrogen. The nitrogen treatment under water deficit conditions showed a six-fold higher expression compared to the treatment NN under water deficit (**Figures 5A, C**).

## 4 Discussion

Most of the plants inoculated with the strains of rhizobacteria evaluated increased root production even under water deficit conditions (**Figure 1**). Similar responses have been reported in other crops such as maize (Kavamura et al., 2013), sorghum (Santana et al., 2020), cowpea (Abiala et al., 2023), and spinach (Jabborova et al., 2023). Many rhizospheric microorganisms are efficient in producing phytohormones such as abscisic acid, gibberellins, cytokinins, but mainly auxins, which promote root growth, branching, and hair formation (Araújo and Hungria, 1999). Among the rhizobacteria evaluated in this study, the production of varying amounts of auxins *in vitro* has already been demonstrated for the strains ESA 13 (Fernandes-Júnior et al., 2015), ESA 402 (Antunes et al., 2019), and ESA 441 (Silva et al., 2018). These factors are likely involved in inducing the increase in the root system of BRS Morena sesame observed in this study. Increased root volume is desirable under water deficit conditions as it allows for greater soil exploration and water absorption. Therefore, the inoculation of bacterial strains that stimulate root growth under water deficit conditions is desirable, and strains with this characteristic are potential inoculants.

The treatments with different inoculants showed an increase in phosphorus content (**Figure 2B**). Walia et al. (2014), in a study with tomatoes using biofertilizers containing *B. subtilis*, observed a 57% increase in phosphorus content. According to these authors, *B. subtilis* has a high capacity to improve plant growth and yield by enhancing the root’s ability to mobilize and absorb nutrients and substances for the overall reproductive fitness of the plants.

The total free proline content in sesame plants on the 5^th^ day of water deficit showed a significant increase in all treatments (**Figure 3A**). This elevated proline content suggests that the plants were under stress and were able to promote osmotic adjustment. Proline is known to play a role in stabilizing proteins, membranes, and cellular structures in plants subjected to stress and may be a factor that restricts the level of Reactive Oxygen Species (ROS). Zhou et al. (2022) investigated sweet potato plants under water stress and found proline accumulation associated with positive regulation of SOD and CAT activities. These characteristics help plants cope with drought (Ebrahimi et al., 2016; Cruz et al., 2022). Like other abiotic stresses, drought results in the accumulation of ROS, primarily in chloroplasts and to some extent in mitochondria. This leads to oxidative stress associated with superoxide anion radical (O_2_^−^) reactions. Oxidative stress can be detoxified by various antioxidants that alleviate oxidative damage and thus confer drought resistance. For example, the activation of the enzymes superoxide dismutase and catalase serves this function (Santos et al., 2022; Lima et al., 2023).

The increase in catalase activity (**Figure 3C**) highlights the importance of antioxidants as a defense mechanism in response to worsening water deficit, preventing oxidative stress (Nikolaeva et al., 2017). According to the results of this research, there may have been the activation of defense systems in sesame, as the enzymes of the plant’s antioxidative mechanism are present in different cellular compartments and effectively contribute to the control of ROS, ensuring redox homeostasis in the system. Among these enzymes, catalase stands out, as it converts H_2_O_2_ to H_2_O and O_2_ without the need for a reducing agent to remove H_2_O_2_, which can favor greater efficiency in the removal of high concentrations of H_2_O_2_ under severe stress (Sharma et al., 2012).

During the research, the increase in SOD activity observed in sesame plants inoculated with growth-promoting bacteria, especially ESA 441 (**Figure 3B**), under water stress conditions is consistent with findings reported by Gholinezhad et al. (2020) in sesame associated with mycorrhizal inoculation under water restriction. These observations can be explained by the fact that plants can overcome the adverse effects of drought through various physiological responses, such as the increase in the activities of antioxidant enzymes to eliminate ROS.

The inoculation with pant001 significantly increased the chlorophyll content in sesame leaves (**Figure 4**), which may be related to the *B. subtilis* capacity to make nitrogen available to plants, a relatively unexplored aspect. In line with this hypothesis, this study observed that pant001 promoted a considerably high increase in nitrogen content (**Figure 2A**). According to López et al. (2014), the chlorophyll content in leaves is directly related to the nitrogen concentration in the plant. Lima et al. (2023) obtained similar results regarding chlorophyll content with the use of *Bacillus* in sesame cultivation. Inoculation with *B. subtilis* also increased chlorophyll a and total chlorophyll concentrations by 30% and 24% in sugarcane plants grown under normal water conditions and by 25% and 29% in plants stressed by drought, respectively (Fonseca et al., 2022). Satapute et al. (2012) and Hashem et al. (2019) suggested that *B. subtilis* can be exploited as a soil inoculant and used to provide nitrogen to soils. Chlorophylls are responsible for absorbing and converting light radiation into energy (ATP and NADPH) in the biological cycle of plants (Larcher, 2000). Therefore, the higher concentration of these pigments in plants subjected to water deficit represents greater stress tolerance capacity due to the intrinsic relationship between chlorophylls, photosynthetic potential, and productivity (O’Neill et al., 2006). Thus, it is expected that inoculation with bacilli can lead to good results in sesame crop productivity since the production of assimilates will be higher.

With the complete sequencing of the sesame genome, many transcription factors involved in responses to biotic and abiotic stresses have been identified, including DREB (Dossa et al., 2016a) and HDZ7, proteins that play crucial roles in gene expression regulation in plants (Mehmood et al., 2022).

In this study, the *DREB* gene profile was analyzed in the BRS Morena sesame genotype. As shown in **Figure 5**, transcript expression was higher in treatments subjected to water stress, which is consistent with findings in the literature reporting the involvement of this gene in plant responses to water stress situations (Guttikonda et al., 2014; Dossa et al., 2016a). According to Zhang and Xia (2023), *DREB* plays a crucial role in plant responses to water deficit. When plants are exposed to water scarcity, *DREB* is activated and binds to specific DNA sequences called Dehydration-Responsive Elements (DRE) to control the expression of genes involved in drought tolerance, activating defense responses so that plants implement a series of mechanisms to cope with water stress. Through the regulation of target genes, *DREB* activates the synthesis of cellular protection proteins, including antioxidant enzymes, membrane-stabilizing proteins, and osmotic regulators (Dossa et al., 2016a). Therefore, the increase in SOD activity (**Figure 3B**) as a plant antioxidant response to the imposed stress may be related to the increased expression of *DREB*.

Moreover, *HDZ7* demonstrated responsivity in most treatments subjected to water deficit in BRS Morena (**Figure 5**). According to the literature, *HDZ7* plays an important role in responding to abiotic stresses, primarily regulating genes related to responses to water deficit, salinity, temperature, light, and other stresses. These target genes may be related to the synthesis of protective proteins, osmotic adjustment, water transport, and other physiological processes that help plants tolerate water stress (Gong et al., 2019; Wei et al., 2019; Baek et al., 2023). Therefore, the BRS Morena sesame genotype, under conditions of water deficit, activates a defense system that includes the increased expression of at least two genes responsive to this condition, *DREB* and *HDZ7*, which in turn activate other defense responses in plants, as observed in this study.

Moreover, it has been observed that *HDZ7* plays a relevant role in root system growth and development. It regulates root architecture, including length, branching, and distribution of root hairs, which can promote the ability of plants to absorb nutrients and water (Wang et al., 2022). The increase in the mass of dry roots in sesame plants subjected to water deficit (**Figure 1B**) may be related to the considerable increase in the expression of *HDZ7* observed in this study. Plants likely benefited from the positive influence of rhizobacteria, as the inoculated treatments promoted an increase in the mass of dry roots. The HD-Zip gene family may become a valuable tool for improving stress tolerance in molecular breeding methods (Wang et al., 2022).

## 5 Conclusion

In this study, it was reported that rhizobacteria can improve the tolerance of sesame plants to water deficit. The results revealed that inoculation positively altered nutritional, biochemical, and vegetative growth parameters of plants subjected to water deficit and plants under normal hydration conditions.

Another noteworthy observation pertained to the defense against ROS, with treatments under water stress showing an increase in both SOD activity and the content of free proline. Some inoculated treatments favored the increase in photosynthetic pigments during water stress. These cascade effects may have potentiated root development. In another line of defense, the *DREB1* and *HDZ7* genes had their expression increased, improving the performance of sesame plants. These results have positive implications for sesame cultivation, especially when grown during periods of reduced water availability, and indicate that the inoculation of rhizobacteria could be an option to mitigate the negative effects of drought using new microbial inoculants.

## Data availability statement

The original contributions presented in the study are included in the article/supplementary materials, further inquiries can be directed to the corresponding author.

## Author contributions

AS: Formal Analysis, Investigation, Writing – original draft. GR: Formal Analysis, Investigation, Validation, Writing – review & editing. AM: Writing – review & editing, Visualization. PF: Visualization, Methodology, Writing – review & editing. NA: Writing – review & editing, Funding acquisition. TG: Writing – review & editing, Conceptualization, Data curation, Methodology. LL: Conceptualization, Funding acquisition, Project administration, Validation, Writing – original draft.
